# Exercise rapidly alters proteomes in mice following spinal cord demyelination

**DOI:** 10.1038/s41598-021-86593-5

**Published:** 2021-03-31

**Authors:** Brian Mark Lozinski, Luiz Gustavo Nogueira de Almeida, Claudia Silva, Yifei Dong, Dennis Brown, Sameeksha Chopra, V. Wee Yong, Antoine Dufour

**Affiliations:** 1 Department of Clinical Neurosciences, University of Calgary, Alberta, Canada; 2 Department of Biochemistry and Molecular Biology, University of Calgary, Alberta, Canada; 3 HRIC 3C64, Department of Physiology and Pharmacology, University of Calgary, 3330 Hospital Drive Calgary, Alberta, T2N 4N1 Canada; 4 The Hotchkiss Brain Institute, University of Calgary, Alberta, Canada; 5 McCaig Institute for Bone and Joint Health, University of Calgary, Alberta, Canada

**Keywords:** Neuroscience, Systems biology

## Abstract

Exercise affords broad benefits for people with multiple sclerosis (PwMS) including less fatigue, depression, and improved cognition. In animal models of multiple sclerosis (MS), exercise has been shown to improve remyelination, decrease blood–brain barrier permeability and reduce leukocyte infiltration. Despite these benefits many PwMS refrain from engaging in physical activity. This barrier to participation in exercise may be overcome by uncovering and describing the mechanisms by which exercise promotes beneficial changes in the central nervous system (CNS). Here, we show that acute bouts of exercise in mice profoundly alters the proteome in demyelinating lesions. Following lysolecithin induced demyelination of the ventral spinal cord, mice were given immediate access to a running wheel for 4 days. Lesioned spinal cords and peripheral blood serum were then subjected to tandem mass tag labeling shotgun proteomics workflow to identify alteration in protein levels. We identified 86 significantly upregulated and 85 downregulated proteins in the lesioned spinal cord as well as 14 significantly upregulated and 11 downregulated proteins in the serum following acute exercise. Altered pathways following exercise in demyelinated mice include oxidative stress response, metabolism and transmission across chemical synapses. Similar acute bout of exercise in naïve mice also changed several proteins in the serum and spinal cord, including those for metabolism and anti-oxidant responses. Improving our understanding of the mechanisms and duration of activity required to influence the injured CNS should motivate PwMS and other conditions to embrace exercise as part of their therapy to manage CNS disability.

## Introduction

Multiple sclerosis (MS) is an inflammatory neurodegenerative disorder in which immune cells enter the central nervous system (CNS) and destroy myelin and myelin-producing oligodendrocytes, as well as neurons and axons^1^. Studies in people with MS (PwMS) have found that exercise improves outcomes of physical fitness such as fatigue and mobility, as well as CNS related outcomes such as cognition and white matter integrity^2–4^. Physical activity performed in a regimented fashion with the purpose of improving physical fitness delineates exercise from physical activity itself^3^. The prior consensus surrounding exercise for PwMS was that their well-being would suffer a detrimental effect due to heat sensitivity and potential aggravation of symptoms^5^. However, as recent studies demonstrate immunomodulatory and clinical benefits of exercise, there is a paradigm shift that now considers exercise as a potential adjunctive therapy for MS and other neurological diseases^3,6^, or even as a preventative therapy for those at risk for developing MS^7^. In the inflammatory experimental autoimmune encephalomyelitis (EAE) rodent model of MS, exercise has the potential to delay disease onset and decrease severity of disability^8–11^.

Exercise can benefit PwMS in many ways. For example, it has peripheral immunomodulatory effects and promotes the release of anti-inflammatory myokines from the contracting muscle, and it also induces regulatory anti-inflammatory microglia activity within the CNS^12–14^. Exercise also promotes neurogenesis and gliogenesis through mechanisms that include the elevation of neurotrophic factors^1,15–18^. Moreover, exercise in mice facilitates remyelination following lysolecithin-induced spinal cord demyelination^19^. Interestingly, exercise was found to work synergistically with clemastine, a known pro-remyelinating drug, to further increase remyelination^19^. The concept of exercise as a co-therapeutic administered with other medications has been termed “MedXercise”, by combining the terms *Med*ication and e*Xercise* (reviewed elsewhere^20^), and may promote further recovery.

Acute bouts of exercise can modulate thousands of molecules in serum samples from human subjects^21^. For instance, it can rapidly increase the blood levels of myeloperoxidase (MPO), which is known to recruit macrophages to damaged sites, revealing a potential benefit for PwMS^21,22^. Additionally, it can increase the levels of neuroactive metabolites, such as acetylcholine and kynurenic acid, linking acute exercise to mental health and antidepressant activity^21,23^. However, little is known about the molecular players capable of bridging the effects of acute and chronic responses to exercise.

Despite the apparent benefits of exercise in MS and rodent MS models, PwMS engage in significantly less physical activity than the healthy population^24^. Recent consensus for activity levels for PwMS by a group of experts in the field recommend encouraging at least 150 min per week^25^. However, besides ambulatory symptoms, the lack of mechanistic understandings on how the CNS is affected by exercise has hampered efforts to motivate PwMS to exercise^3^. The available evidence from human and animal studies is inconclusive regarding questions such as when exercise should be initiated in relation to relapses, how much is required, and what intensity or paradigm will produce the greatest benefit.

Here, we profiled the spinal cord and serum of mice using quantitative shotgun proteomics to address whether short bouts of exercise may influence the peripheral blood and CNS of naïve animals and following LPC-induced spinal cord demyelination. Our approach has the potential to identify changes at the micro-environment or systemic level, by analyzing the spinal cord and serum, respectively. We demonstrate that access to voluntary running wheel activity over four days is sufficient to induce significant protein level changes within the lesioned spinal cords associated with oxidative stress, metabolism, neurotransmission and proteolytic remodeling of the extracellular matrix. These results provide insight into the impact of exercise on demyelinating injuries.

## Material and methods

### Animals

All experiments were conducted with ethics approval from the Animal Care Committee at the University of Calgary under regulations of the Canadian Council of Animal Care. All mice used were female C57BL/6 mice at 6–12 weeks of age acquired from Charles River (Montreal, Canada). Mice were between 18–21 g in body weight and were maintained on a 12-h light/dark cycle with food (Pico-Vac Mouse Diet 20) and water given ad libitum.

### Lysolecithin (lysophosphatidylcholine)-induced demyelination

Demyelination was accomplished as previously described^26^. Mice were anaesthetized using ketamine (100 mg/kg) and xylazine (10 mg/kg) injected intraperitoneally. Skin overlying surgical site was shaved and disinfected with 70% ethanol and iodine. Ophthalmic gel was applied to both eyes to prevent drying, and buprenorphine (0.05 mg/kg) was injected subcutaneously immediately prior to surgery and 12 h post-surgery as an analgesic. Animals were positioned on a stereotaxic frame and a midline incision approximately 5 cm long was made between the shoulder blades using a #15 scalpel blade. A retractor was used to separate the muscle and adipose tissue to expose the spinal column. The prominent T2 vertebra was used as a landmark to find the T3–T4 intervertebral space. Tissue in the T3–T4 gap was then blunt dissected apart using forceps and spring scissors, and the dura was removed using a 30-gauge metal needle. Using a 32-gauge needle attached to a 10 µL Hamilton syringe, 0.5 µL of 1% lysolecithin/lysophosphatidylcholine (LPC) (Sigma-Aldrich, L1381) was injected into the ventral column of the spinal cord at a rate of 0.25 µL/min for 2 min. The needle was left in place for 2 min following the injection to avoid back flow, followed by suturing of the muscle and skin. Mice were then placed in a thermally controlled environment for recovery.

### Exercise paradigm

Immediately following recovery from surgery (~ 1–2 h post-surgery), exercising animals were singly housed in modified rat cages^19^ with 5 inch running wheels mounted to the wire lid for 4 days. Wheels were connected to a computer system running a software to monitor wheel revolutions (developed in LabVIEW). Rotations per minute (rpm) were recorded in 10-min bins generating an average rpm over that 10-min period. Sedentary control animals were singly housed in modified rat cages with the running wheel placed on the ground as environmental enrichment control. Naïve animals were given access to running wheel cages at the same time as LPC animals.

### Serum and tissue collection for proteomics

For the proteomics experiment, serum and lesioned spinal cords were collected from eight different female C57BL6 mice per group and two mice were pooled per sample: naïve without exercise (n = 4), naïve with exercise (n = 4), LPC lesioned without exercise (n = 4) and LPC lesioned with exercise (n = 4). Tissue was harvested 4 h into the dark cycle when animals had reached peak running for 2 h. Mice were anaesthetized using ketamine (100 mg/kg) and xylazine (10 mg/kg) injected intraperitoneally, and 500 µL of whole blood was drawn from the heart using a 1 mL syringe with a 25-gauge needle and collected in a 1.5 mL microcentrifuge tube. Mice were then transcardially perfused with 15 mL of room temperature phosphate-buffered saline (PBS) solution. Spinal tissue from the lower cervical to lower thoracic region was dissected. Tissue was placed on a dissecting microscope set up with dry ice and 2-methylbutane (Sigma-Aldrich) placed underneath the stage allowing for the tissue to be frozen to avoid protein degradation. Spinal cords were trimmed to 5 mm around the lesion site then cut into quarter-sections to include the lesion area. Lesioned quarter-sections from 2 mice were pooled into one sample in a 1.5 mL microcentrifuge tube to allow for sufficient protein levels and left on dry ice until harvesting was finished. Tissue was stored at − 80 °C until the proteomics experiments were performed. Tissue collection for immunohistochemistry followed a different methodology described below.

Whole blood was spun at 1300 rpm (160 g) for 10 min at 4 °C. Supernatant was removed using a pipette being careful to avoid disrupting the red blood cell pellet. As in spinal cords, serum from 2 animals from the same group were pooled, for a final n = 4 sample (from 8 mice) per group.

### Quantitative shotgun proteomics using tandem mass tags (TMT) labeling

The four groups of mice were subjected to a quantitative shotgun proteomics analysis (Supplementary Tables 1–4). Serum or tissue were lysed in a buffer composed of 1% SDS, 200 mM HEPES (pH 8.0), 100 mM ammonium bicarbonate, 10 mM EDTA and protease inhibitor cOmplete tablets (Roche, 4693159001). Disulfide bonds of 100 μg of total protein were reduced with 10 mM Tris(2-carboxyethyl)phosphine hydrochloride (Thermo Fisher Scientific) at 55 °C for 1 h. The proteins were then alkylated by incubation with 15 mM iodoacetamide (VWR) for 25 min in the dark at room temperature. Proteins were precipitated out of solution by adding 600 μL of ice-cold acetone and incubated at − 20 °C overnight. Samples were centrifuged at 8000*g* for 10 min before resuspension in 100 μL of 50 mM triethyl ammonium bicarbonate. Proteins were then trypsinized (Thermo Fisher Scientific) overnight at a 1:10 enzyme-to-substrate ratio. For TMT 6-plex labeling, 0.8 mg of TMT reagent (Thermo Fisher Scientific) was resuspended in 41 μL of acetonitrile (ACN), samples were spun down quickly at 2000 rpm (380*g*) for 10 s, and samples were incubated at room temperature for 1 h. The labelling reaction was quenched by adding 8 μL of 5% hydroxylamine and incubated for 15 min at 25 °C. Peptides with different labels were combined before 100% formic acid was added to each sample to reach a volumetric concentration of 1% formic acid. Samples were spun at 5000 rpm (2350*g*) for 10 min and then desalted using Sep-Pak C18 columns (Waters, 130 mg WAT023501). Sep-Pak columns were conditioned with 1 × 3 mL 90% methanol/0.1% TFA, 1 × 2 mL 0.1% formic acid. Each sample was loaded onto a column and washed with 1 × 3 mL 0.1% TFA/5% methanol. Peptides were eluted off the column with 1 × 1 mL 50% ACN/0.1% formic acid and lyophilized. Peptides were resuspended in 1% formic acid and a BCA assay (Thermo Fisher Scientific) was used to determine the concentration of peptide in each sample. Samples were dried down and stored at − 80 °C.

### High performance liquid chromatography (HPLC) and mass spectrometry

All liquid chromatography and mass spectrometry experiments were carried out by the Southern Alberta Mass Spectrometry (SAMS) core facility at the University of Calgary, Canada. To assure consistency in data collection, HPLC and mass spectrometry data acquisition was replicated from our previous publication^27^. In detail, analysis was performed on an Orbitrap Fusion Lumos Tribrid mass spectrometer (Thermo Fisher Scientific) operated with Xcalibur (version 4.0.21.10) and coupled to a Thermo Scientific Easy-nLC (nanoflow Liquid Chromatography) 1200 system. Tryptic peptides (2 μg) were loaded onto a C18 trap (75 μm × 2 cm; Acclaim PepMap 100, P/N 164946; Thermo Fisher Scientific) at a flow rate of 2 μL/min of solvent A (0.1% formic acid and 3% acetonitrile in LC- mass spectrometry grade water). Peptides were eluted using a 120 min gradient from 5 to 40% (5% to 28% in 105 min followed by an increase to 40% B in 15 min) of solvent B (0.1% formic acid in 80% LC- mass spectrometry grade acetonitrile) at a flow rate of 0.3 μL/min and separated on a C18 analytical column (75 um × 50 cm; PepMap RSLC C18; P/N ES803; Thermo Fisher Scientific). Peptides were then electrosprayed using 2.3 kV voltage into the ion transfer tube (300 °C) of the Orbitrap Lumos operating in positive mode. The Orbitrap first performed a full mass spectrometry scan at a resolution of 120,000 FWHM to detect the precursor ion having a mass-to-charge ratio (m/z) between 375 and 1575 and a + 2 to + 4 charge. The Orbitrap AGC (Auto Gain Control) and the maximum injection time were set at 4e5 and 50 ms, respectively. The Orbitrap was operated using the top speed mode with a 3 s cycle time for precursor selection. The most intense precursor ions presenting a peptidic isotopic profile and having an intensity threshold of at least 2e4 were isolated using the quadrupole (isolation window of m/z 0.7) and fragmented with HCD (38% collision energy) in the ion routing Multipole. The fragment ions (MS2) were analyzed in the Orbitrap at a resolution of 15,000. The AGC, the maximum injection time and the first mass were set at 1e5, 105 ms and 100, respectively. Dynamic exclusion was enabled for 45 s to avoid of the acquisition of same precursor ion having a similar m/z (plus or minus 10 ppm).

### Proteomic data and bioinformatics analysis

Spectral data were matched to peptide sequences in the murine UniProt protein database using the Andromeda algorithm^28^ as implemented in the MaxQuant^29^ software package v.1.6.0.1, at a peptide-spectrum match false discovery rate (FDR) of < 0.01. Search parameters included a mass tolerance of 20 p.p.m. for the parent ion, 0.5 Da for the fragment ion, carbamidomethylation of cysteine residues (+ 57.021464 Da), variable N-terminal modification by acetylation (+ 42.010565 Da), and variable methionine oxidation (+ 15.994915 Da). TMT 6-plex labels 126–131 were defined as labels for relative quantification. The cleavage site specificity was set to Trypsin/P (search for free N-terminus and for only lysines), with up to two missed cleavages allowed. Next, quantified proteins were filtered using the Perseus software^30^. Proteins that have more than two replicates with zero values in more than one group were removed. Filtered proteins were normalized using the CycLoess approach via NormalizerDE package^31^ using the R language (v3.6.0)^32^. An average of the normalized results for each group was calculated and followed by the ratio of each group comparison. The ratios were log(2) transformed and the significant outlier cut-off values were determined after log(2) transformation by boxplot-and-whiskers analysis using the BoxPlotR tool^33^.

### Heatmaps

The results from the BoxplotR analysis were used in the heatmaps. Data analysis was accomplished using the R software^32^. The plot was generated using the heatmap.2 function from the gplots package^34^.

### Reactome pathway analysis

To identify protein–protein interaction, the STRING (Search Tool for the Retrieval of Interacting Genes) database^35^ was used to illustrate interconnectivity among proteins. Protein interaction relationship is encoded into networks in the STRING v11 database (https://string-db.org). Our data was analyzed using the *Mus musculus* as our model organism at a false discovery rate of 5%. Proteins belonging to specific pathways were selected and their ratios were plotted as heatmaps.

### Immunohistochemistry

For histology, lesioned spinal cords were collected from five different female C57BL6 mice per group: LPC lesioned without exercise (n = 5) and LPC lesioned with exercise (n = 5). The experiment was performed in duplicates, for a total of twenty mice. Tissue was harvested 4 h into the dark cycle when animals had reached peak running for 2 h. Mice were transcardially perfused with 15 mL of room temperature PBS followed by 15 mL of ice cold 4% paraformaldehyde (PFA). The spinal cord was dissected from the lower cervical to lower thoracic regions and post fixed in 4% PFA overnight at 4 °C, then cryoprotected in 30% sucrose for 3 days. Cords were cut into 5 mm sections with the lesion site in the centre and frozen in FSC 22 Frozen Section Media (Leica). Spinal cords were cut coronally into 20 μm sections on a cryostat (Thermo Fischer Scientific) onto Superfrost Plus microscope slides (VWR). Sections were stored at − 20 °C until staining and analysis. Slides with coronal sections were warmed to room temperature for 5 min. For myelin basic protein (MBP) staining, slides were delipidated with concurrent washes of 50%, 70%, 90%, 95%, 100%, 95%, 90%, 70%, and 50% ethanol. Slides were rehydrated for 10 min in PBS, then permeabilized with 0.2% Triton-X100 in PBS for 10 min. Slides were then blocked for 1 h at room temperature using horse blocking buffer (PBS, 10% horse serum, 1% BSA, 0.1% cold fish stain gelation, 0.1% Triton X-100, 0.05% Tween-20) and IgG Fab fragments (20 µg/mL). Staining for NFH, Olig2, PDGFRα, CC, and CX32 did not require delipidating steps. After blocking, primary antibodies were added in antibody dilution buffer (PBS, 1% BSA, 0.1% cold fish stain gelation, 0.1% Triton X-100) overnight at 4 °C. Sections were washed three times with 0.05% Tween-20 in PBS for 5 min each, followed by 1 h of incubation with secondary antibodies in antibody dilution buffer (1/400 from manufacturer stock) and Nuclear Yellow (1/800) at room temperature. Sections were then washed three more times with 0.05% Tween-20 in PBS for 5 min each then mounted using Fluoromount-G solution (SouthernBiotech). The following antibodies were used: Olig2 (Millipore AB9610, R&D AF 2418), PDGFRα (R&D AF1062), CC1 (Millipore OP80-100UG), MBP (Abcam AB7349), NFH (Encor RPCA-NFH), GFAP (Biolegend 829401), GJB1 (Invitrogen 71-0600). The following Jackson laboratory secondary antibodies were used: Cyanine Cy3 donkey anti-chicken IgY, Cyanine Cy3 donkey anti-rat IgG, Alexa Fluor 594 donkey anti-mouse IgG, Alexa Fluor 488 donkey anti-rat IgG, Alexa Fluor 647 donkey anti-rat IgG, Alexa Fluor 657 donkey anti-rabbit IgG.

### Confocal microscopy

Confocal images were taken on the Leica TCS Sp8 laser confocal microscope using the 10 × air objective and 25 × 0.5 NA water objective. The 405 nm, 488 nm, 552 nm, and 640 nm lasers were used to excite fluorophores with detection by 2 low dark current Hamamatsu PMT detectors and two high sensitivity hybrid detectors. Images were acquired in 8-bits, in a z-stack bidirectionally, with 2-times line average, and at 0.57 μm optical sections. Laser levels were maintained across all sections of common stains, and secondary alone controls were used to account for non-specific secondary fluorescence. Images were blinded and analyzed using ImageJ (RRID: SCR_003070).

### Statistical analysis

For sample size calculation, it was used previously published studies as a reference^19^, using as a baseline the least sensitive method. The Shapiro–Wilk test was used to assess data normality and the *F*-test was used to evaluate if the variances of two groups are equal. Normal data were evaluated using the Student *t*-test, while non-normal data was assessed using the Mann–Whitney test. For the proteomics analysis, it was applied an interquartile boxplot analysis to determine the proteins differentially expressed^33^. The FDR was generated by the bioinformatic tools used for pathway analysis. The R software^34^, GraphPad Prism version 9, and Microsoft Excel were used for the statistical analysis.

### Ethical standards

Our study was carried out in compliance with the ARRIVE guidelines.

## Results

### Exercise in naïve mice alters the spinal cord proteome

**Figure 1:**
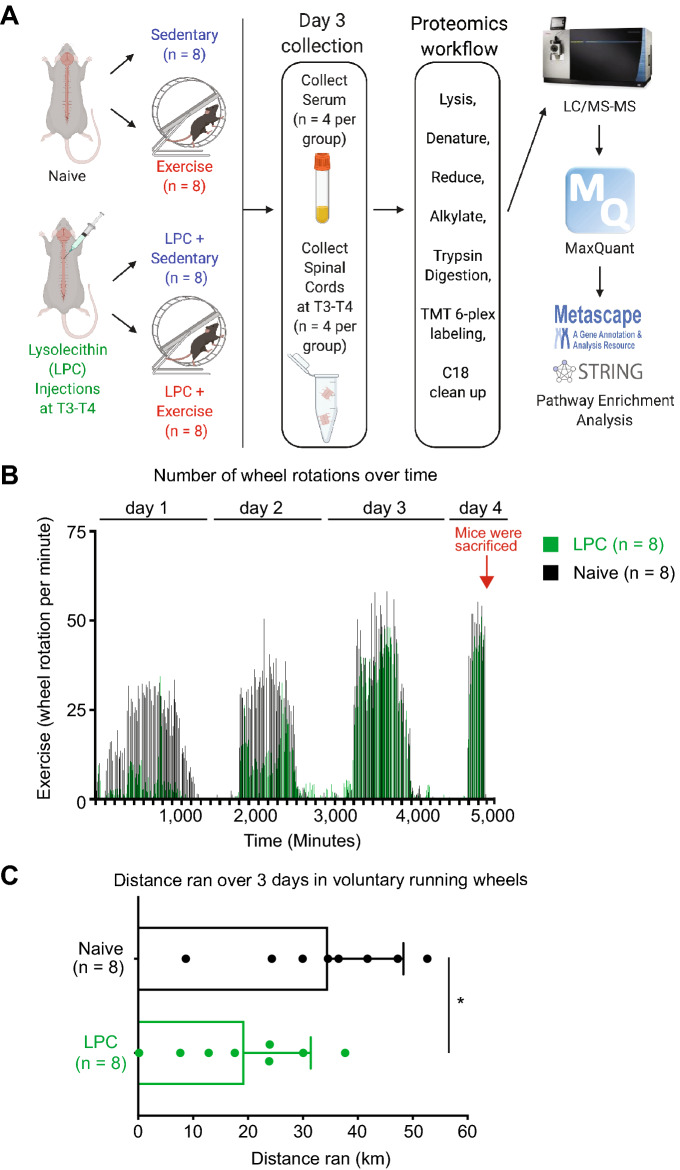
Workflow and running wheel activity. The LPC mice recovered for ~ 1–2 h post-surgery and the same rest period was given to naïve mice. Naïve and LPC animals were given access to a running wheel, or a wheel on its side immediately following recovery. (**A**) Schematic demonstrating experimental workflow. Animals were subjected to LPC surgery or not, then housed with an unlocked or locked running wheel. Serum and tissue were collected on the 4th day (4 h into the running cycle) and subjected to a quantitative shotgun proteomics workflow. Spinal cords and serum were pooled in groups of 2 for adequate protein concentrations (n = 4 of 2 pooled samples, n = 8 total). Figure was drawn using BioRender. The data was analyzed using the freely available MaxQuant software v.1.6.0.1 (https://www.maxquant.org). (**B**) Running wheel data of naïve and LPC mice. Revolutions were monitored and binned in 10-min increments. Figure was drawn using Prism. (**C**) Quantification of total distance run over a 4-day period for naïve (n = 8) and LPC (n = 8) mice. Student’s *t*-test was used for statistics. *P < *0.05*.

To evaluate the impact of exercise on the CNS and serum, we subjected mice to short-term bouts of exercise and compared to sedentary control. Running animals had free access to a running wheel over 4 days, while sedentary animals were given a locked wheel as environmental enrichment control. Mice ran during their night cycle and rested during the day as determined from the number of runs per 10 min bin (Fig. 1A,B). Four hours into day 4 and during the animal’s active night cycle, serum and spinal cords at T3–T4 were collected and subjected to a quantitative shotgun proteomics workflow labelled with tandem mass tags (TMT) 6-plex kits (Fig. 1A). Mice subjected to LPC-surgery had reduced exercise activity in the first and second day after surgery but attained similar levels by the end of the third day (Fig. 1B); over the 4 days, LPC-surgery mice ran significantly shorter distances than the naïve animals (Fig. 1C).

**Figure 2:**
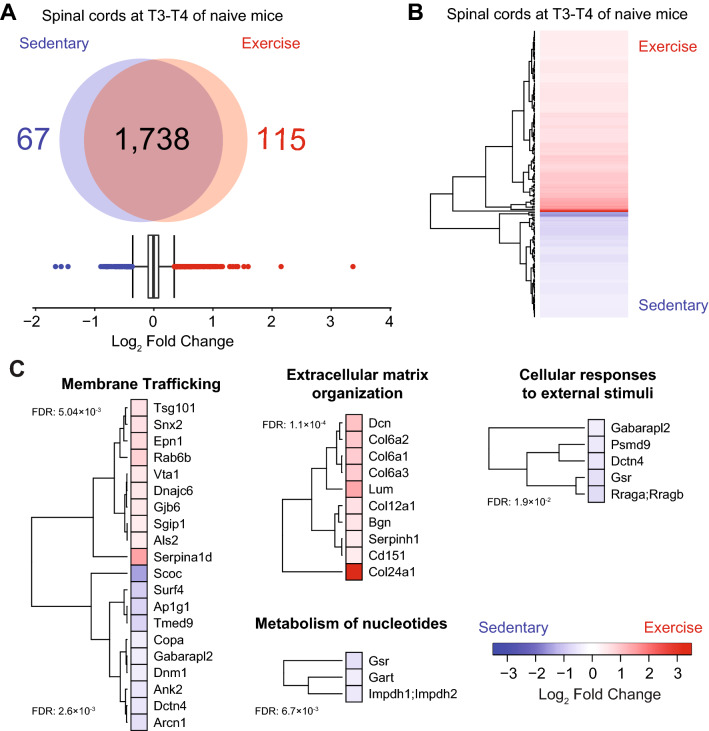
Effect of exercise on naïve spinal cord proteome after 4 days of running. Red, significantly elevated in the exercise group; Blue, significantly elevated in the sedentary group. (**A**) Quantification of differentially expressed proteins as determined by interquartile box plot analysis. A false discovery rate (FDR) of 1% was applied to the database search on MaxQuant. (**B**) Heatmap of differentially expressed proteins in naïve spinal cord tissue. (**C**) Heatmap of proteins and associated reactome pathways as determined by STRING (https://string-db.org). A false discovery rate (FDR) for each pathway was added on the left of each heatmap. Interquartile box plot analysis was used for statistics. Data analysis was accomplished using the R software^32^. The plot was generated using the heatmap.2 function from the gplots package^34^.

We first examined protein changes in non-lesioned naïve mice that exercised on the running wheel to determine if short term exercise could alter the CNS proteome. Compared to their sedentary naïve controls, there were 115 significantly upregulated proteins and 67 significantly downregulated proteins in the spinal cord following exercise as determined by an interquartile boxplot analysis (Fig. 2A,B; Supplementary Tables 1–2). Pathway enrichment analyses were performed using STRING^35^ and Metascape^36^ and revealed various altered pathways (Fig. 2C and Supplementary Figure 1). Upregulated proteins in the CNS included those associated with membrane trafficking (e.g. Snx2: sorting nexin-2), the extracellular matrix (e.g. Bgn: biglycan, Col’s: collagens) (Fig. 2C), glutathione anti-oxidant responses (e.g. Mgst1: microsomal glutathione S-transferase 1, Gstk1: glutathione S-transferase kappa 1), metabolism (e.g. Acsl1: long chain fatty acid CoA ligase 1, Mgll: monoglyceride lipase) and myelination (Mbp: myelin basic protein, Myrf: myelin regulatory factor) (Supplementary Figure 1A,C). Downregulated proteins in exercising mice (i.e. higher in sedentary mice) included those related to metabolism of nucleotides (e.g. Gart: Trifunctional purine biosynthetic protein adenosine-3) (Fig. 2C) and Golgi-ER transport (e.g. Copa: Coatomer submit alpha, Dctn4: dynactin subunit 4) (Supplementary Figure 1B,C).

In the serum of naïve exercising animals there were 9 upregulated proteins including those related to detoxification of oxygen species (Gpx3: glutathione peroxidase 3, Prdx2: peroxiredoxin-1), and platelet degranulation (Fgb: fibrinogen beta chain, Ecm1: extracellular matrix protein 1) (Supplementary Figure 2A,B,D and Supplementary Tables 3–4). Exercising naïve mice had 11 downregulated proteins (i.e. higher in sedentary mice) including those associated with plasma lipoprotein assembly (ApoA4: Apolipoprotein A-4, ApoB: Apolipoprotein B-100) (Supplementary Figure 2A,C,D). Our findings suggest that acute exercise in naïve mice can alter both the CNS and serum proteomes. Proteins associated with anti-oxidant responses are concurrently elevated in spinal cord and serum of naïve exercising mice.

### Demyelination following LPC injection

**Figure 3:**
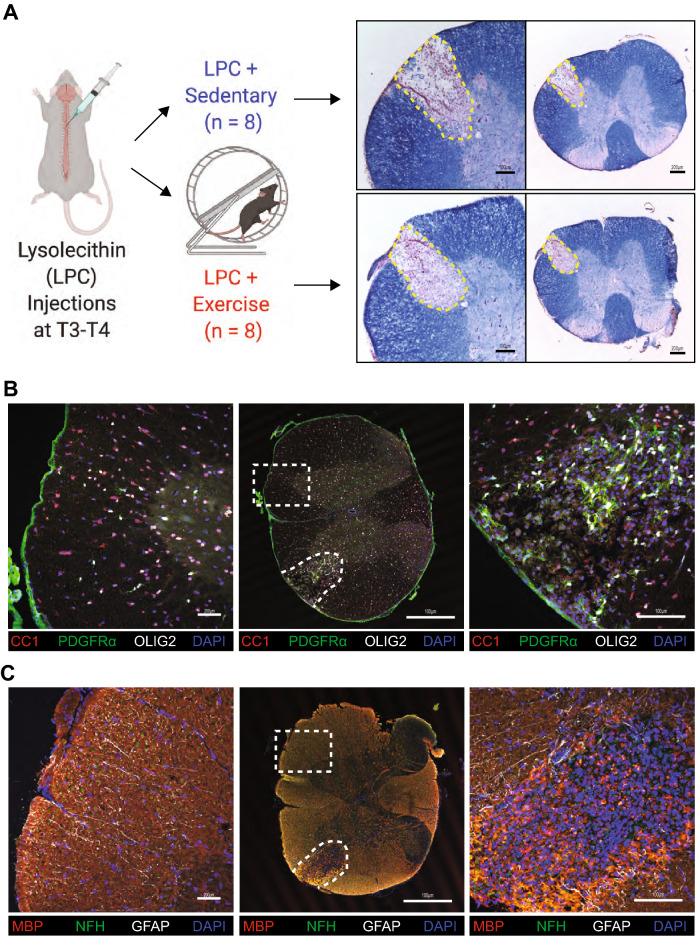
Impact of LPC surgery on mice subjected to running wheel. (**A**) Workflow and representative eriochrome cyanine-stained sections with lesion in the ventrolateral white matter delineated by dashed lines. Figure was drawn using BioRender. (**B**) Representative images of LPC lesion (left) and contralateral normal appearing white matter (right) 4 days post injury stained for mature oligodendrocytes (CC1) in red, OPCs (PDGFRα) in green, oligodendrocyte lineage cells (OLIG2) in white, and DNA (DAPI) in blue. (**C**) Representative images of LPC lesion 4 days post injury stained for myelin and myelin debris (MBP) in red, axons (NFH) in green, and astrocytes (GFAP) in white, and DNA (DAPI) in blue. In both (**B**) and (**C**), the lesion is outlined by the irregular dashed line while the non-involved contralateral site is denoted by the rectangle dashed line. Scale bar represents 100 μm.

Next, we determined whether demyelinated CNS also have altered proteomes in response to exercise. To model the environment of a demyelinated MS lesion, we injected LPC into the spinal cords of mice^26^ and compared to naïve uninjured mice. LPC-injected mice were placed in individual cages with a running wheel immediately after recovery from surgery in the LPC group. Following LPC injection, there was demyelination as verified by eriochrome cyanine staining, and a loss of mature OLIG2 + CC1 + oligodendrocytes within the lesion; the number of OLIG2 + PDGFRα + oligodendrocyte progenitor cells (OPC) in the lesion appeared increased at 3 days post lesion (dpi) compared to the contralateral uninjured side (Fig. 3A,B). There was increased detection of MBP positive myelin debris, visible loss of axons, and a lack of GFAP positive astrocytes within the lesion (Fig. 3C). These results affirm the creation of a demyelinated lesion against which exercise in previous experiments ^19^ promoted oligodendrogenesis at later time points, and they provide the substrate to evaluate the proteomics of exercise following demyelination.

### Wheel running induces significant protein changes within the lesioned spinal cord

**Figure 4:**
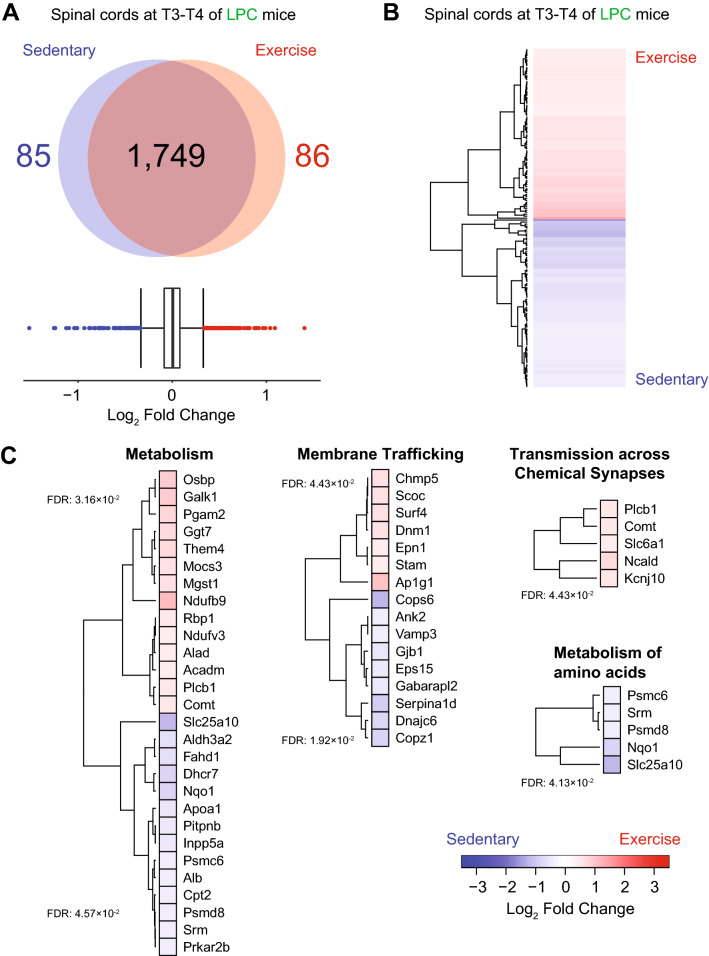
Effect of exercise on LPC demyelinated spinal cord proteome after 4 days of running wheel. (**A**) Quantification of differentially expressed proteins in LPC demyelinated spinal cord 4 dpi as determined by interquartile box plot analysis. A false discovery rate (FDR) of 1% was applied to the database search on MaxQuant. (**B**) Heatmap of differentially expressed proteins in LPC demyelinated spinal cord. **C**) Heatmap of proteins and associated reactome pathways as determined by STRING (https://string-db.org). A false discovery rate (FDR) for each pathway was added on the left of each heatmap. Interquartile box plot analysis was used for statistics. Data analysis was accomplished using the R software^32^. The plot was generated using the heatmap.2 function from the gplots package^34^.

**Figure 5:**
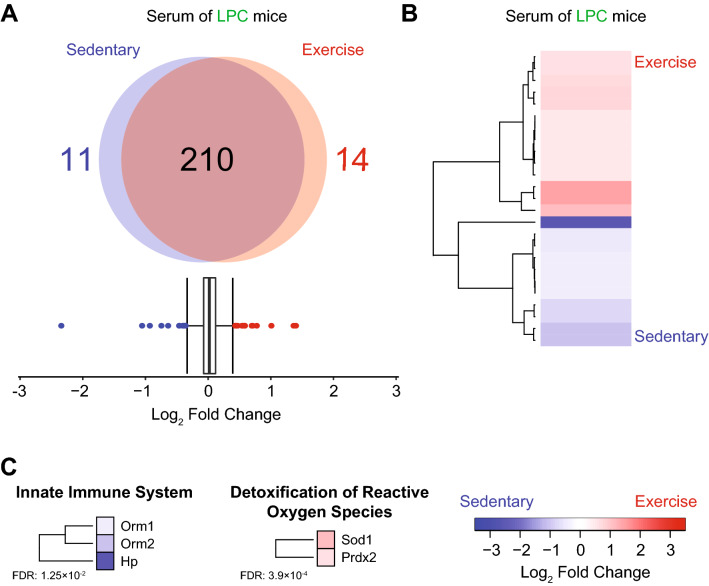
Effect of exercise on LPC demyelinated serum proteome after 4 days of running wheel. (**A**) Quantification of differentially expressed proteins in LPC demyelinated serum 4 dpi as determined by interquartile box plot analysis. A false discovery rate (FDR) of 1% was applied to the database search on MaxQuant. (**B**) Heatmap of differentially expressed proteins in LPC demyelinated serum. (**C**) Heatmap of proteins and associated reactome pathways as determined by STRING (https://string-db.org). A false discovery rate (FDR) for each pathway was added on the left of each heatmap. Interquartile box plot analysis was used for statistics. Data analysis was accomplished using the R software^32^. The plot was generated using the heatmap.2 function from the gplots package^34^.

We identified 1920 proteins within the spinal cord of LPC demyelinated mice, and 248 proteins in the serum (Figs. 4A,B, 5A). Of the identified proteins, 86 were significantly upregulated and 85 were significantly downregulated in the lesioned spinal cords of exercising mice compared to sedentary demyelinated controls (Fig. 4A,B and Supplementary Tables 1–2); 14 were upregulated and 11 downregulated in the serum (Fig. 5A,B and Supplementary Tables 3–4).

Compared to sedentary demyelinated mice, wheel running following demyelination increased proteins associated with metabolism (e.g. Ndufb9: NADH dehydrogenase [ubiquinone] 1 beta subcomplex subunit 9, Pgam2: phosphoglycerate mutase 2), glutathione anti-oxidant responses (Mgst1: microsomal glutathione S-transferase 1), transmission across chemical synapse (e.g. Ncald: neurocalcin delta) and proteolytic activity (e.g. ADAM10: a disintegrin and metalloprotease 10, Serpin1b: leukocyte elastase inhibitor B) (Fig. 4C, Supplementary Figure 3). Conversely, exercise decreased proteins (i.e. elevated in sedentary over exercise spinal cords) associated with glucose metabolism (Gnpda1: Glucosamine-6-phosphate isomerase 1, Slc25a10: mitochondrial phosphate carrier protein); the gap junction protein, connexin 32 (Gjb1, gap junction beta1 protein), and MBP and Myrf were also reduced in exercising demyelinated samples (Fig. 4C, Supplementary Figure 3).

In the serum of exercising animals, proteins associated with the innate immune system were downregulated (Orm1: Alpha-1-acid glycoprotein-1, Orm2), and anti-oxidant proteins (Sod1: superoxide dismutase 1, Prdx2: peroxiredoxin-2) were upregulated (Fig. 5C, Supplementary Figure 4). This shows that acute amounts of exercise are sufficient to profoundly alter the proteome of the spinal cord and serum in both naïve and LPC demyelinated animals.

### Proteomes commonly elevated by exercise in naïve and demyelinated spinal cords

**Figure 6:**
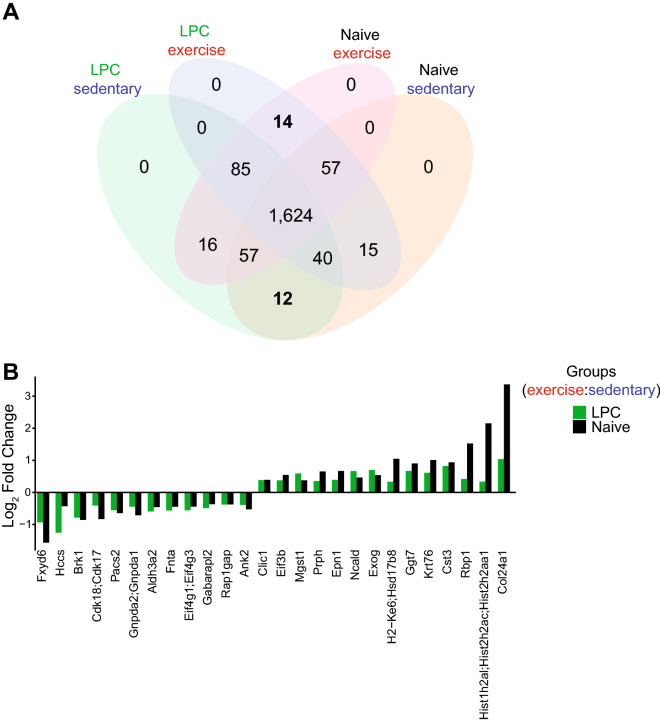
Comparison of shared proteins from the naïve and LPC of sedentary and exercising spinal cord proteome. (**A**) Venn diagram of enriched proteins in each of the 4 experimental groups after boxplot analysis. A total of 1624 proteins were not statistically enriched in any of the groups. 14 common upregulated and 12 common downregulated proteins between naïve and LPC exercising animals are shown in bold. (**B**) Bar plot showing the 14 proteins upregulated and the 12 proteins downregulated in both the naïve and LPC mice. Data analysis was accomplished using the R software^32^. The plot was generated using the heatmap.2 function from the gplots package^34^.

We addressed whether there were proteins commonly elevated by exercise in naïve and LPC-demyelinated spinal cords. The Venn diagram in Fig. 6A shows 14 proteins elevated by exercise in both the LPC and naïve animals including those associated with oxidative stress response (Mgst1: microsomal glutathione S-transferase 1, Ggt7: gamma-glutamyl transferase 7 involved in the metabolism of glutathione), extracellular matrix (Col24a1: collagen alpha-1 chain), protease activity (Cst3: cystatin-C), transcription/translation (Eif3b: Eukaryotic translation initiation factor 3 subunit B, Hist1h2al: a histone) and metabolism (Rbp1: retinol-binding protein) (Fig. 6B). Others elevated by exercise are Clic1 (Chloride intracellular channel protein 1), Epin1 (epsin-1 associated with endocytosis), Exog (a mitochondria nuclease), Ncald (Neuron-specific calcium-binding protein hippocalcin), and Prph (peripherin/vimentin).

Finally, twelve proteins were commonly downregulated by exercise in the spinal cord of naïve and LPC mice (Fig. 6B). They include Fxyd6 (FXYD domain-containing ion transport regulator 6), Hccs (cytochrome c-type heme lyase), Brk1 (protein BRICK 1), Cdk17/18 (cyclin-dependent kinase-17 or 18), Pacs2 (phosphofurin acidic cluster sorting protein 2), Gnpda1/2 (glucosamine-6-phosphate isomerase 1 or 2), Aldh3a2 (aldehyde dehydrogenase), Fnta (protein farnesyltransferase), Eif4g1/3 (eukaryotic translation initiation factor 4 gamma 1 or 3), Gabarapl2 (gamma-aminobutyric acid receptor-associated protein-like 2), Rap1gap (Rap1 GTPase-activating protein 1) and Ank2 (ankyrin-2).

### Validation by immunofluorescence microscopy of decrease of connexin-32 and myelin basic protein in 4-day lesion of exercising animals

**Figure 7:**
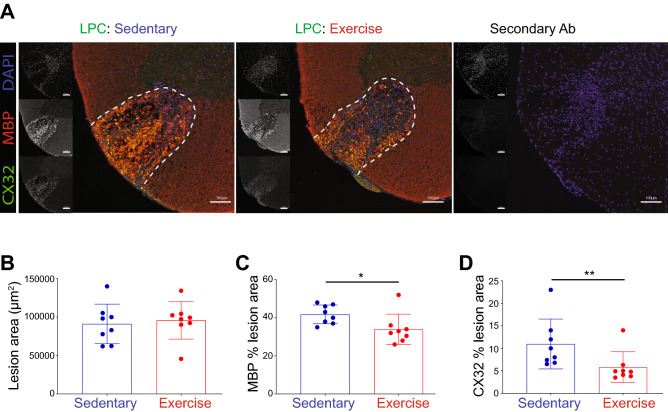
Immunofluorescence microscopy of LPC mice validation of shotgun proteomics analysis. (**A**) Representative images of LPC mice from the sedentary control, exercising animals, and secondary control for connexin-32 (CX32), and myelin (MBP). Scale bar denotes 100 μm. (**B**) Quantification of lesion area. (**C**) Quantification of percent of lesion that is MBP-positive. *p < 0.05*.* (**D**) Quantification of percent of lesion that is CX32-positive. **p < 0.01. A Mann–Whitney test was used for statistics. Each point is of a single animal, and the bar represents mean ± SD (n of 8 per group).

We sought to validate the findings of proteomics by immunofluorescent staining of coronal spinal cord sections. An antibody each to a marker of metabolism (Pgam2) and anti-oxidant enzyme (Mgst1) was first attempted but signals of immunofluorescence were unconvincing in any sections. We next chose antibodies to structural proteins such as the gap junction protein, connexin-32, and MBP, which have been pointed out above to be reduced in exercising demyelinated mice. Using immunofluorescence, we found that while lesion size at 4 days post-LPC did not differ between exercising and sedentary groups (Fig. 7A,B), there was a significant decrease in the amount of both connexin-32 and MBP immunoreactivity within the lesion environment of exercising animals (Fig. 7C,D). As MBP immunoreactivity within the LPC lesion at early time points post-injury (and which is devoid of eriochrome cyanine as in Fig. 3) is indicative of myelin debris^37^, the decrease in MBP in lesion of exercising mice may represent the more rapid clearance of myelin debris that is inhibitory to repair processes.

## Discussion

There is unanimity that exercise benefits general health. For brain health, several reports demonstrate that exercise modulates the brain’s connectome and even its structure^38–40^. There is now an extensive literature that exercise improves cognition, fatigue, depression and mood in PwMS^3,7^. In animal studies, outcomes of exercise in healthy mice or those modeling neurological conditions include reduced neuroinflammation or blood–brain barrier breakdown, neurogenesis, oligodendrogenesis, neuroprotection and remyelination^1,15–18^. The mechanisms by which exercise promotes CNS wellbeing appear to be multiple, such as the generation of brain-derived neurotrophic factor, reducing pro-inflammatory responses, modulating microglia activity, and ameliorating oxidative stress (reviewed in^12,14,41,42^). Endurance (treadmill running) training in rodents decreased oxidative stress in the spinal cord, while increasing levels of the Nrf2 transcription factor and downstream anti-oxidant enzymes^43^. Exercise also promotes neurotransmission and multiple signaling pathways in brain cells^17,42^. Molecular mediators of the benefits of exercise include muscle-derived myokine FNDC5/irisin that prevents neurodegeneration and rescues memory impairment in a model of Alzheimer’s disease, and liver-derived glycosylphosphatidylinositol-specific phospholipase D1 that improves cognition in aged mice^44,45^.

**Figure 8:**
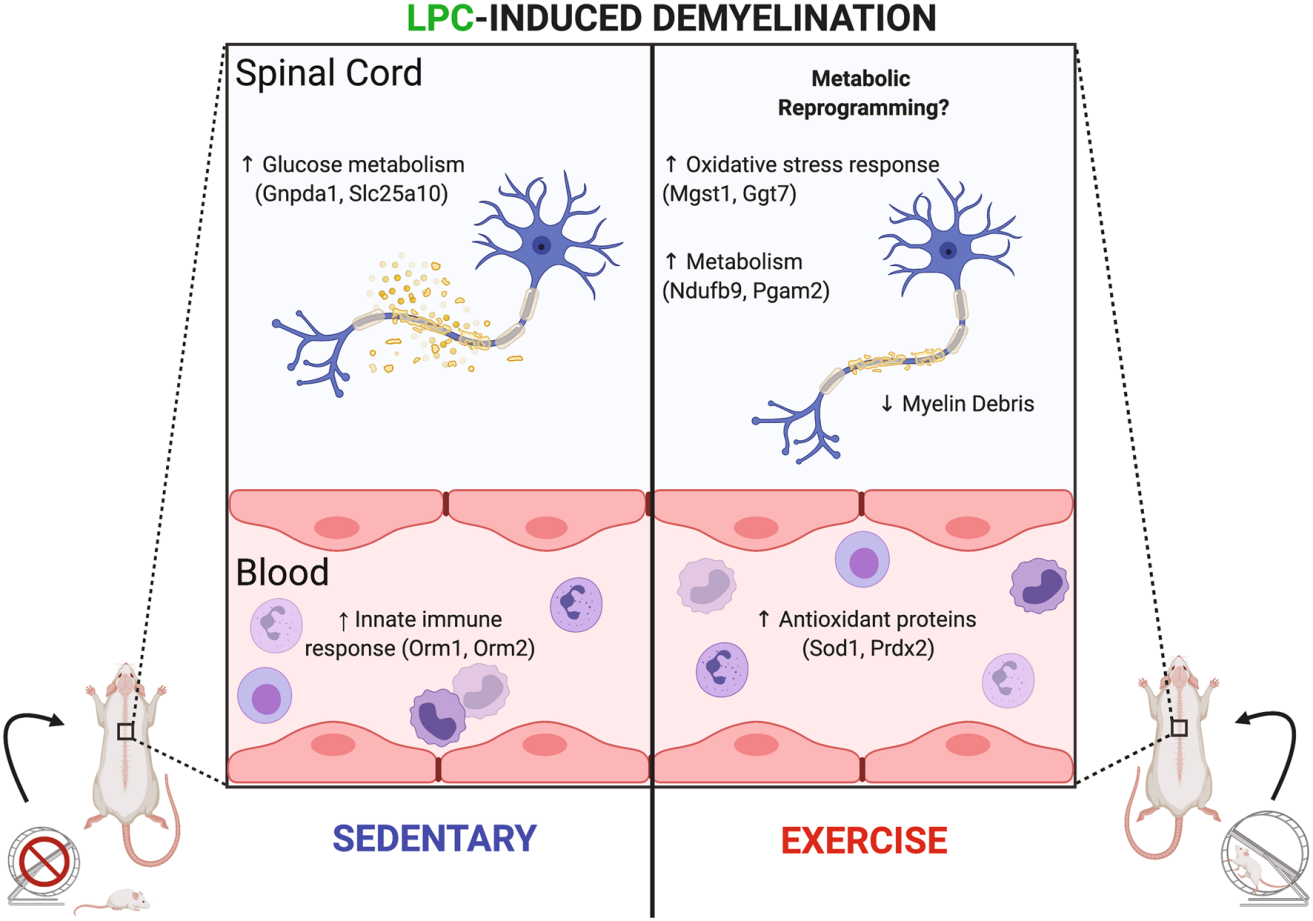
Schematic representation of key protein changes between naïve and LPC mice that underwent 4 days of exercise or remained sedentary. Figure was drawn using BioRender.

Despite the increasing knowledge of the mechanisms of exercise, it is not known how quickly they are affected. Here, using quantitative shotgun proteomics, we report that a short bout of exercise changes numerous proteins in blood and spinal cord of naïve and demyelinated mice (Fig. 8). Running activity of demyelinated mice was low to modest in the first 2 days and was similar to naïve controls only by the fourth day. Thus, the proteomic changes from tissues harvested 4 h into running at day 4 (Fig. 1) are reasonably the result of less than 2 days of running. Unanswered questions are whether the proteomic changes would be affected after a single running episode, or whether some would be long-lasting after exercise is stopped. Regardless, with a short bout of running, the results are remarkable, as over 150 proteins are up- or down-regulated in the spinal cord of both naïve and demyelinated mice. Fewer altered proteins are detected in the serum, likely the result of our technical detection of a smaller number of proteins (248) in the serum compared to the spinal cord (1920 proteins) of LPC demyelinated mice (Figs. 4A, 5A).

The quick alteration of numerous proteins in the CNS may help explain the brain changes in humans soon after exercise. For example, a single 50-min vigorous intensity cycling on a stationary bicycle in healthy subjects improved their prefrontal cortex-dependent cognition that lasted up to 2 h after exercise^46^. A single 40-min bout of harness-supported treadmill walking in highly progressed PwMS increases brain excitability, which indicates higher potential for neuroplasticity, and it is more pronounced in individuals with higher aerobic fitness. Although this feature was observed only on the hemisphere less affected by the disease, it shows the importance of exercise in PwMS and the potential for changes in the CNS even following acute exercise^47^.

In evaluating the changes to the proteomes in the spinal cord of demyelinated mice after exercise, we found that markers of anti-oxidant responses, metabolism, neurotransmission and proteolytic remodeling of the extracellular matrix are commonly elevated by exercise. The last is notable since subsequent repair after injury would require extracellular matrix remodeling^48^. Elevation of anti-oxidant responses are also notable. Markers of oxidative stress are increased within MS lesions where they have been implicated in axonal damage and demyelination^1,49,50^. In the EAE model of MS, the primary anti-oxidant glutathione is upregulated following voluntary wheel running^51^. Consistent with this report, we found elevation of Mgst1 and Ggt7 in the exercising LPC-demyelinated spinal cord. Mgst1 has both glutathione transferase and peroxidase functions which makes it particularly potent at attenuating oxidative stress injuries^52,53^. Thus, exercise-induced upregulation of these antioxidants may help mitigate injury-related oxidative damage. We attempted to corroborate these findings by looking at markers of oxidative injury such as malondialdehyde (MDA), 8-Oxo-2′-deoxyguanosine (8-OhDG), and 4-hydroxynonenal (4-HNE). Due to limitations with high antibody background staining and thus specificity we were unable to corroborate differences in overall oxidative stress. However, we hypothesize that any difference would likely be seen later in lifespan of the lesion after these oxidative stress response proteins have had time to function. We also found increased expression of Sod1 and Prdx2 in the serum of exercising mice. Decreased total antioxidant capacity has been noted in the serum of PwMS^54,55^. Taken together, the combined spinal cord and serum upregulation of antioxidants suggests that exercise produces the necessary components to handle the pro-oxidants commonly seen in MS.

Changes in metabolism influence cell function^56,57^. Metabolic reprogramming such as the reliance of aerobic glycolysis for rapid energy supply occurs in cells when responding to injury and infection^57–59^. For example, macrophages rely on aerobic glycolysis to transmigrate into the parenchyma from perivascular cuffs in the EAE model^60^. Additionally, microglia are thought to use oxidative phosphorylation for homeostatic or tissue repair functions^61,62^ but switch to aerobic glycolysis when pro-inflammatory^63^. Our proteomic analysis identified significant changes in metabolism-related proteins. For example, upregulation of Pgam2 suggests a general rise in glycolysis while increased expression of proteins associated with mitochondria (Ndufb9, Ndufv3, mt-Nd3) suggests elevated oxidative phosphorylation. These metabolic changes could be downstream of the transcriptional co-activator PGC1α (peroxisome proliferator-activated receptor gamma coactivator 1-alpha), which mediates exercise-enhanced remyelination in mice^19^.

Impressively, exercise in naïve mice also elevated numerous proteins (115) in the naïve spinal cord of mice, with smaller changes (9 elevated) noted in the serum. This finding may help account for the benefit of exercise not only for general health, but also for CNS wellbeing in the absence of any pathology. Common pathways elevated by exercise in the naïve or demyelinated CNS include those related to anti-oxidants and metabolism. Namely, the upregulation of metabolism upon exercise was identified by 16 and 18 proteins in the LPC (FDR = 3.16 × 10^–2^) and naïve (FDR = 1.29 × 10^–2^) samples, respectively. Among the 34 proteins, they shared only Ggt7, Mgst1 and Rbp1, indicating that exercise has a wide effect on protein expression in the spinal cord tissue, modulating the levels of multiple proteins within a specific biological function. For the proteins commonly enriched in sedentary mice, Fxyd6 had the highest log fold change, -1.56 naïve and -0.93 LPC. Experimental evidence indicates that the FXYD family of transmembrane proteins can associate and modulate the transport properties of Na,K-ATPase^64,65^, an enzyme responsible for regulating the Na^+^ and K^+^ gradients across the cell membrane^66^. Dysfunction of Na,K-ATPase may play a central role in MS, as loss of axonal Na,K-ATPase is linked to neurological decline in chronic stages of the disease^66^. Although the exact impact of Fxyd6 on Na,K-ATPase is not completely known, its regulation by exercise, independently of LPC, suggests a beneficial and protective role for PwMS. These findings may also help explain why MS is less common in those that exercise versus those that do not, supporting the suggestion that exercise may prevent MS^7,67^.

We note that while several pathways have been identified by Metascape and STRING analyses for exercise-induced changes, and several proteins have been highlighted, these proteins may serve several functions in different pathways. For instance, Mgst1 is highlighted in both anti-oxidant and metabolism pathways in our analyses. Also, not all existing proteins have been identified in the serum and spinal cord in this study, a limitation of mass spectrometry, so other exercise-induced changes remain to be discovered.

When comparing the proteomics findings to the transcriptomics results from a previous study published by our group^19^, it is not possible to see a clear correlation between both techniques, even though it was analyzed in the same type of LPC-induced demyelinating lesions. However, this lack of correlation may be explained by the number of days post lesion (dpl) analyzed in each experiment, as the proteomics approach was performed on 3 dpl and the transcriptomics analyzed samples at 5 and 10 dpl. Nonetheless, proteomics identified 4 members of the mitogen-activated protein kinases (Mapk) family (Mapk1, Mapk3, Mapk15, and Mapk10) with minimal alterations in their fold change, while the ERK/MAPK signaling pathway was enriched at 5 dpl on the transcriptomics data and presented the lowest p-value (2 × 10^–5^). This suggests that the MAPK pathway is part of a delayed response induced by exercise and most likely to be detected at the protein level on day 5 and onwards.

LPC demyelination provides a useful model for subsequent remyelination^26^. However, it models the repopulation of oligodendrocyte lineage cells after demyelination in MS and does not replicate every facet of the disease. We acknowledge that this is not a classical immune mediated lesion as is seen in MS or EAE, but rather a toxin induced injury. LPC has been useful because of the well characterized injury and a reliable course of resolution. Following cell death and lesion formation there is a rapid increase in the innate immune response by day 3. Some limitations of this model include the dearth of an adaptive immune response. While lymphocytes have been shown to be present early in lesion formation, the short lifespan and focal nature of the lesion restricts the immune response to an innate one. By day 7 following lesion formation, the repair process has begun and OPCs accumulate in the lesion. By two weeks, remyelination begins to occur and is robust by three weeks. For the purposes of this study, to investigate what changes exercise induces in naïve and demyelinated tissue, LPC is a useful model. LPC produces rapid and complete demyelination within the first day of application^68^, and the eriochrome cyanine staining shows a complete loss of intact myelin at day 4 (Fig. 3A). Nonetheless, myelin debris is still manifest within the lesion at this point, as noted by MBP immunoreactivity (Fig. 7) within the lesion that is intense due presumably to exposure of antibody epitopes in the degraded myelin. In previous work, we found that exercise promotes myelin debris removal by 3–4 days of injury^19^, likely resulting in the lower MBP immunoreactivity in the lesion of exercising mice herein (Fig. 7). This is important in demyelinating injuries in which myelin debris is inhibitory to OPCs^37,69^ and would need to be cleared. However, we did not find phagocytosis-associated proteins to be elevated in the exercise LPC group, but endocytosis-associated proteins were increased (Chmp5: Charged multivesicular body protein 5, Dnm1: Dynamin-1, Ehd1: EH domain-containing protein 1, Epn1: Epsin-1, Git1: ARF GTPase-activating protein GIT1, and Stam: Signal transducing adapter molecule 1) (Supplementary Table 1).

In the lysolecithin-injury, OPCs begin repopulating within 3 days but maturation to oligodendrocytes occurs later (approximately day 7). This may reconcile the result that Myrf, a transcriptional factor expressed in mature myelinating oligodendrocytes that promotes the expression of myelin genes^70^, is downregulated in the LPC spinal cord by exercise, as the day four post-LPC time point is not yet timely for myelin formation. Conversely, Myrf is elevated by exercise in the naïve spinal cord (Supplementary Figure 1), suggestive of activity-dependent myelin formation in the homeostatic CNS^17^.

In summary, we provide a protein level landscape of how exercise alters the CNS. The rapid elevation of numerous proteins involved in several pathways, particularly anti-oxidative and metabolic responses, not only following demyelination but also in the healthy state, is remarkable (Fig. 8). These rapid changes help reconcile the observations that short bouts of exercise can influence the brain. A finer dissection of the crucial proteins may provide direction for future studies of exercise to promote repair responses in MS and other neurological conditions. Finally, the profound changes to the proteome induced by exercise may provide a lesion milieu particularly conducive for a pro-remyelinating medication to act upon. This integration of *Med*ication and e*Xercise* or “MedXercise” may lay the foundation for future strategies to maximize regeneration of the injured CNS.

## Supplementary Information

Supplementary Figures.

Supplementary Tables.

## Data Availability

The proteomics data are publicly available and are deposited in PRIDE Archives, accession number: PXD020782.

## Code availability

The R codes are available upon request.
