# Supplementary material for: Exercise rapidly alters proteomes in mice following spinal cord demyelination
Source: Sci Rep. 2021 Mar 31;11:7239. doi: 10.1038/s41598-021-86593-5 (PMC8012633; doi:10.1038/s41598-021-86593-5)
Supplement: Supplementary file 1 — Supplementary Figures. [file 41598_2021_86593_MOESM1_ESM.pdf]

Supplementary Information for

"Exercise rapidly alters proteomes in mice following spinal cord demyelination"

Brian Lozinski*, Luiz Gustavo Nogueira de Almeida*, Claudia Silva, Yifei Dong, Dennis Brown, Sameeksha Chopra, V. Wee Yong, Antoine Dufour

1- **Supplementary Figure 1**. Significant spinal cord proteins identified by shotgun proteomics and associated enriched pathway changes following exercise in naive mice.

2- **Supplementary Figure 2**. Significant serum proteins identified by shotgun proteomics and associated enriched pathway changes following exercise.

3- **Supplementary Figure 3**. Significant spinal cord proteins identified by shotgun proteomics and associated enriched pathway changes following exercise in LPC mice.

4- **Supplementary Figure 4**. Significant serum proteins identified by shotgun proteomics and associated enriched pathway changes following exercise in LPC mice.

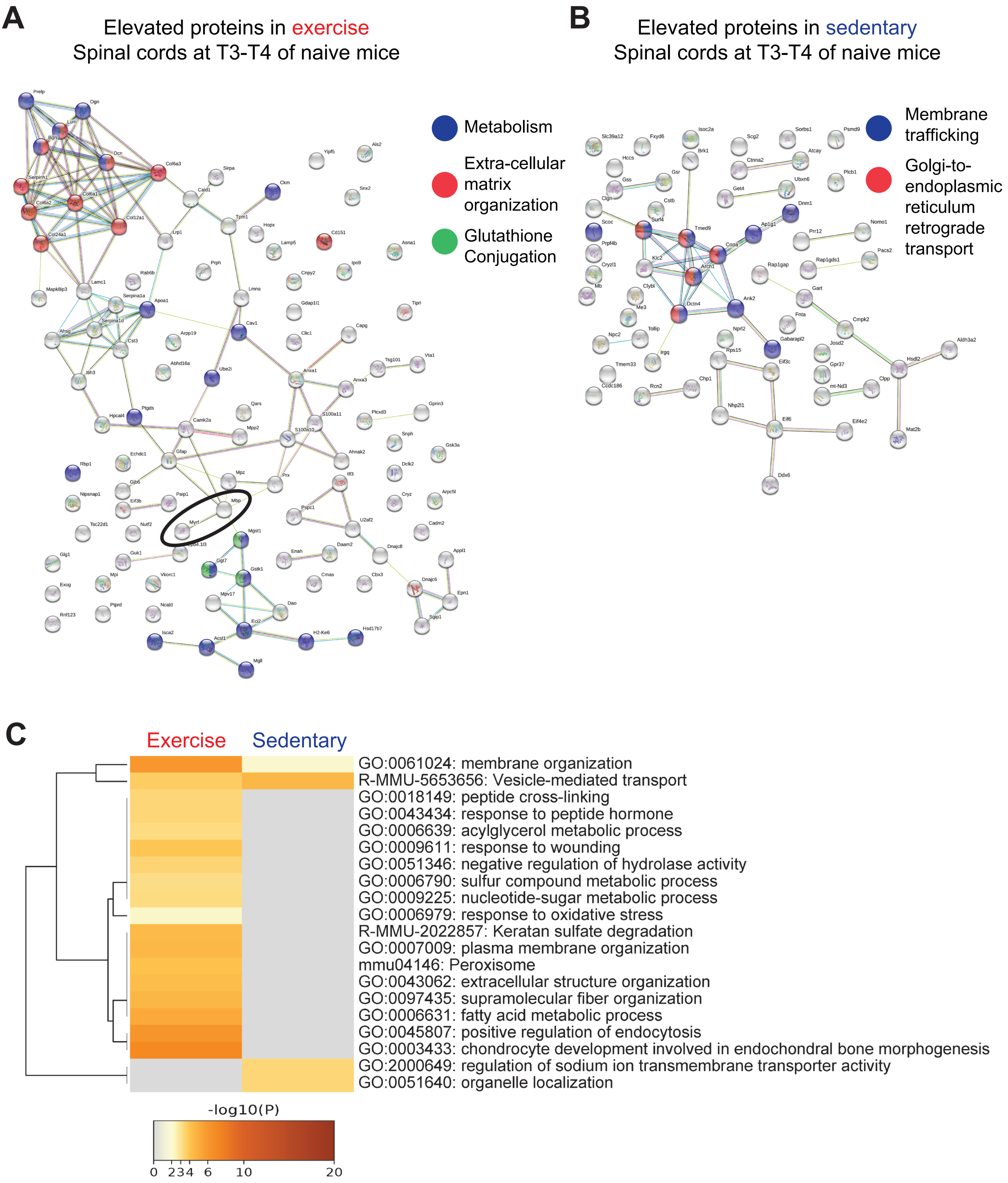

Supplementary Figure 1. Significant altered spinal cord proteins identified by shotgun proteomics and associated enriched pathway changes following exercise in naive mice. STRING-db analysis of proteins significantly changing in the **A**) exercise group and **B**) sedentary group. Significantly changed reactome pathways are colored in each group. $P < 0.05$ as determined by False Discovery rate (FDR). **C**) Metascape analysis of the significantly changed proteins for each group. Terms with a $p < 0.01$ are shown.

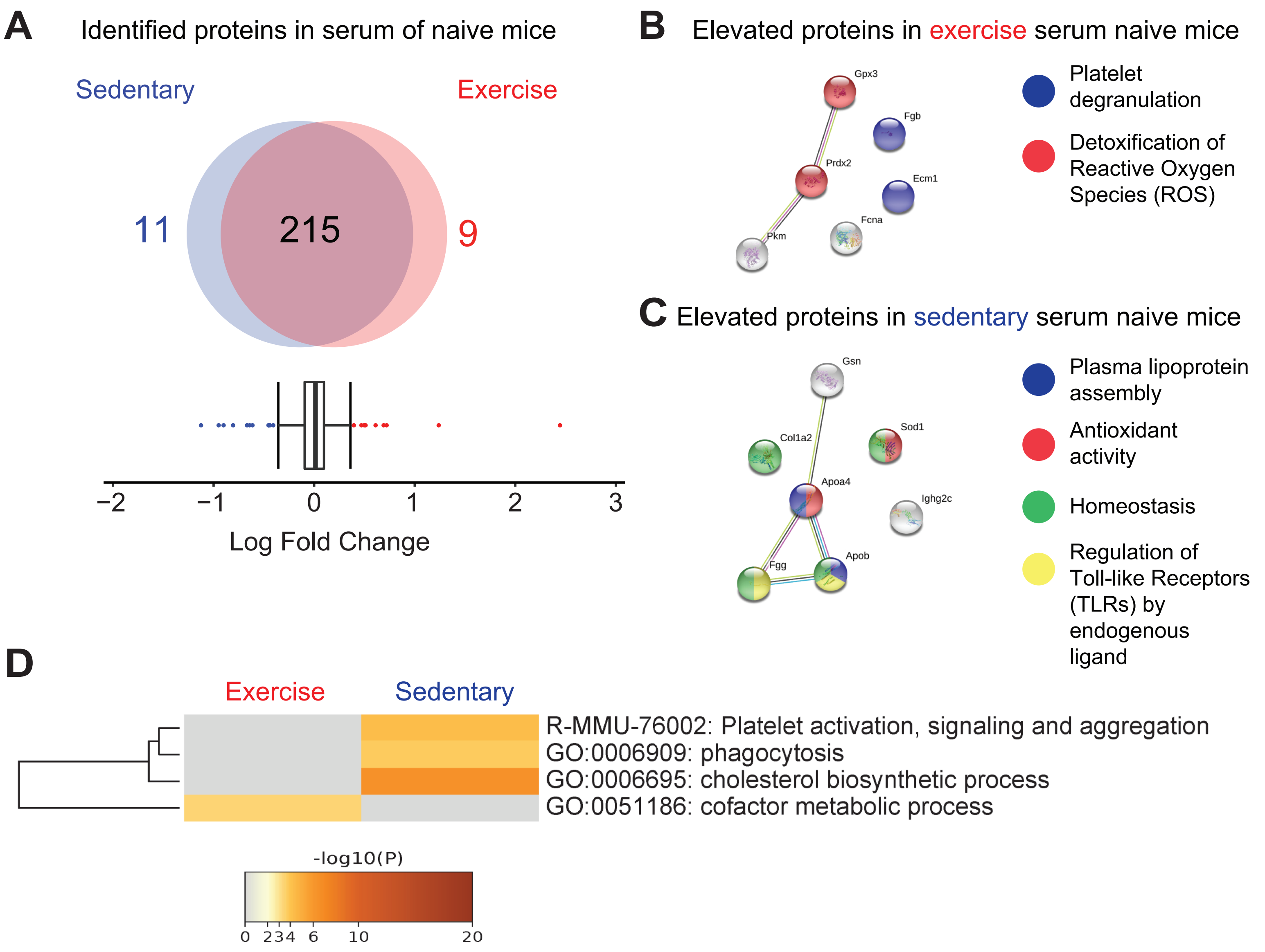

Supplementary Figure 2. Significant altered serum proteins identified by shotgun proteomics and associated enriched pathways changes following exercise in naive mice. **A**) Quantification of significantly changed proteins as determined by interquartile box plot analysis. STRING-db analysis of proteins significantly changing in the **B**) exercise group and **C**) sedentary group. Significantly changed reactome pathways are colored in each group. *P* < 0.05 as determined by False Discovery rate (FDR). **D**) Metascape analysis of the significantly changed proteins for each group. Terms with a *p* < 0.01 are shown.

Supplementary Figure 3 Lozinski*, De Almeida* *et al.*

A Elevated proteins in exercise spinal cords at T3-T4 of LPC mice

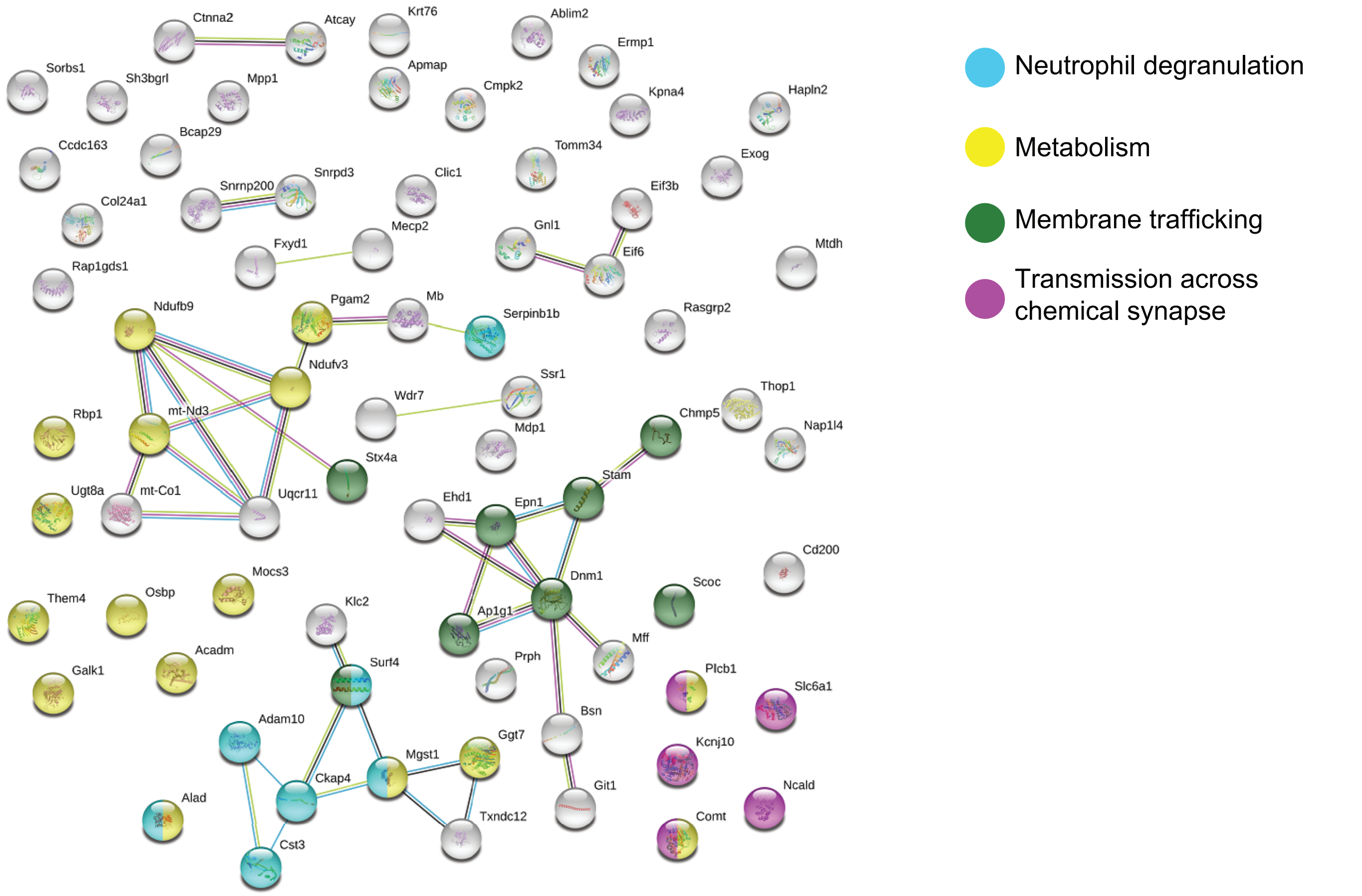

B Elevated proteins in sedentary spinal cords at T3-T4 of LPC mice

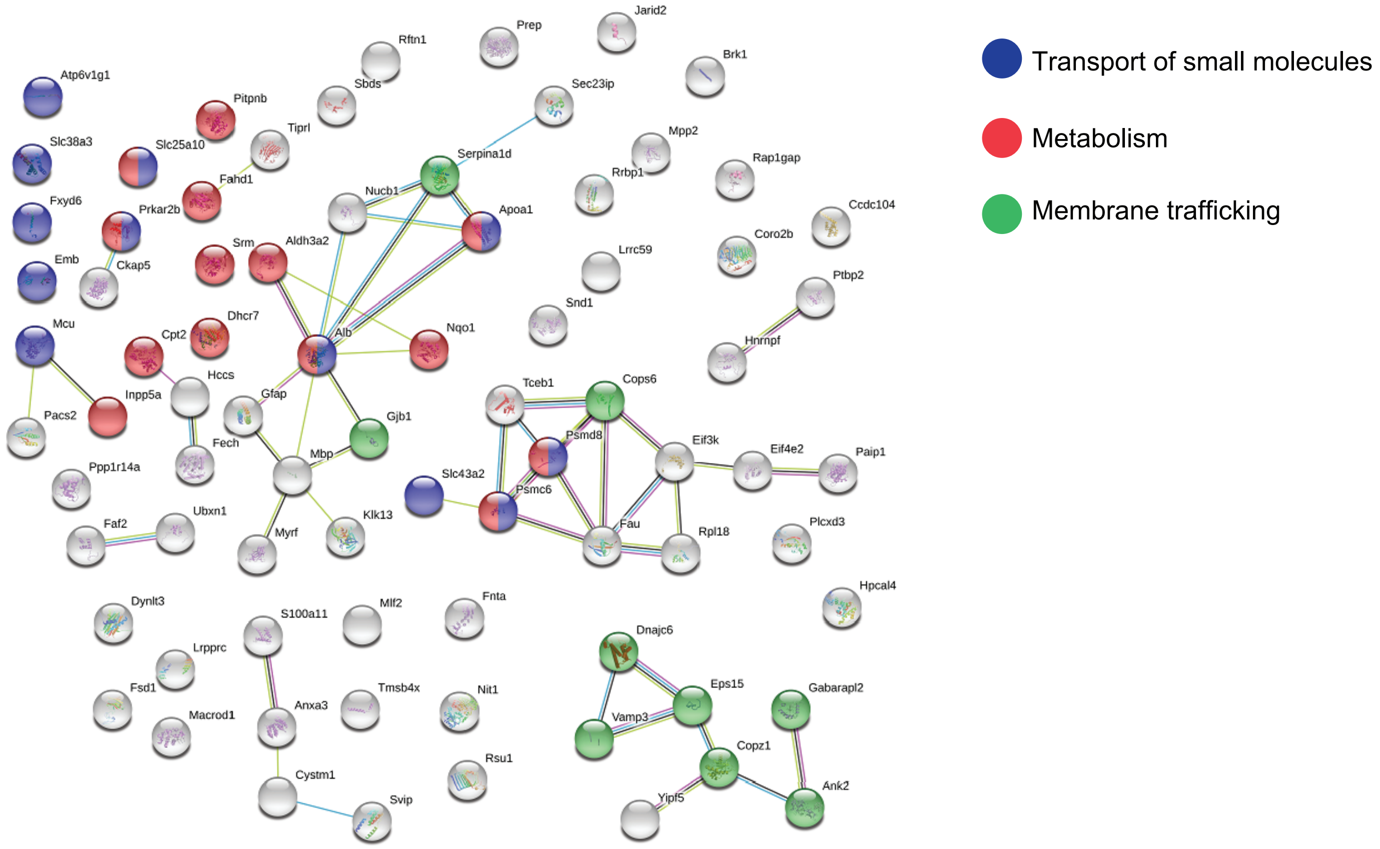

Supplementary Figure 3. Significant altered spinal cord proteins identified by shotgun proteomics and associated enriched pathway changes following exercise in LPC mice. STRING-db analysis of proteins significantly changing in the **A**) exercise group and **B**) sedentary group. Significantly changed reactome pathways are colored in each group. *P* < 0.05 as determined by False Discovery rate (FDR).

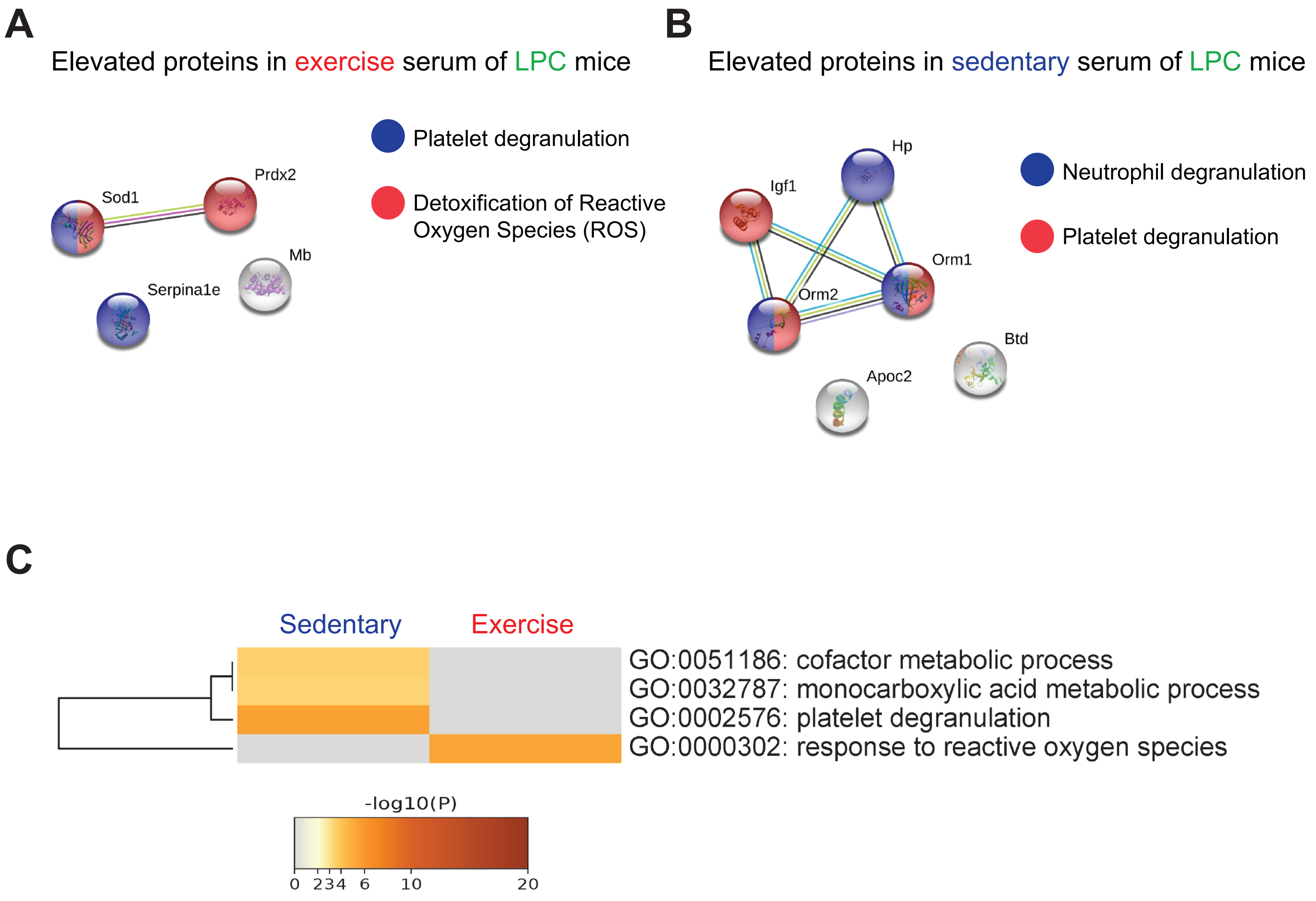

Supplementary Figure 4. Significant altered serum proteins identified by shotgun proteomics and associated enriched pathway changes following exercise in LPC mice. STRING-db analysis of proteins significantly changing in the **A**) exercise group and **B**) sedentary group. Significantly changed reactome pathways are colored in each group. $P < 0.05$ as determined by False Discovery rate (FDR). **C**) Metascape analysis of the significantly changed proteins for each group. Terms with a $p < 0.01$ are shown.
